# Whole-exome sequencing as the first-tier test for patients in neonatal intensive care unit: a Chinese single-center study

**DOI:** 10.1186/s12887-024-04820-0

**Published:** 2024-05-22

**Authors:** Ruiping Zhang, Xiaoyu Cui, Yan Zhang, Huiqing Ma, Jing Gao, Ying Zhang, Jianbo Shu, Chunquan Cai, Yang Liu

**Affiliations:** 1 Present Address: Department of Neonatology, Tianjin Children’s Hospital/Tianjin University Children’s Hospital, Beichen District, Tianjin, China; 2 Graduate College, Tianjin Medical University, Heping District, Tianjin, China; 3 Tianjin Pediatric Research Institute, Tianjin Children’s Hospital/Tianjin University Children’s Hospital, Beichen District, Tianjin, China; 4 Tianjin Key Laboratory of Birth Defects for Prevention and Treatment, Beichen District, Tianjin, China; 5 The Pediatric Clinical College in Tianjin Medical University, Heping District, Tianjin, China

**Keywords:** Genetic disorders, Neonatal intensive care unit, Neonates, Whole-exome sequencing

## Abstract

**Background:**

Genetic disorders significantly affect patients in neonatal intensive care units, where establishing a diagnosis can be challenging through routine tests and supplementary examinations. Whole-exome sequencing offers a molecular-based approach for diagnosing genetic disorders. This study aimed to assess the importance of whole-exome sequencing for neonates in intensive care through a retrospective observational study within a Chinese cohort.

**Methods:**

We gathered data from neonatal patients at Tianjin Children’s Hospital between January 2018 and April 2021. These patients presented with acute illnesses and were suspected of having genetic disorders, which were investigated using whole-exome sequencing. Our retrospective analysis covered clinical data, genetic findings, and the correlation between phenotypes and genetic variations.

**Results:**

The study included 121 neonates. Disorders affected multiple organs or systems, predominantly the metabolic, neurological, and endocrine systems. The detection rate for whole-exome sequencing was 52.9% (64 out of 121 patients), identifying 84 pathogenic or likely pathogenic genetic variants in 64 neonates. These included 13 copy number variations and 71 single-nucleotide variants. The most frequent inheritance pattern was autosomal recessive (57.8%, 37 out of 64), followed by autosomal dominant (29.7%, 19 out of 64). In total, 40 diseases were identified through whole-exome sequencing.

**Conclusion:**

This study underscores the value and clinical utility of whole-exome sequencing as a primary diagnostic tool for neonates in intensive care units with suspected genetic disorders. Whole-exome sequencing not only aids in diagnosis but also offers significant benefits to patients and their families by providing clarity in uncertain diagnostic situations.

**Supplementary Information:**

The online version contains supplementary material available at 10.1186/s12887-024-04820-0.

## Background

Genetic disorders (GDs) are a significant concern in the neonatal intensive care unit (NICU), contributing to approximately 20% of infant deaths [1, 2]. The incidence of GDs diagnoses has risen in recent years, largely due to advancements in genomic sequencing [1, 3]. GDs are diverse and have serious clinical implications, affecting patient diagnoses and their quality of life. Early diagnosis is crucial, benefiting from clinical management and treatment. However, clinical signs are often subtle in the early stages, particularly in newborns in the NICU, where symptoms may not be fully apparent. Sometimes, the phenotype of GDs can be obscured by other clinical symptoms, complicating diagnosis despite numerous routine and specialized tests, including invasive procedures and repeated blood sampling. These processes can cause significant distress to patients, financial strain on families, and yet may not elucidate the underlying pathogenesis [4, 5]. The rapid progression of GDs can lead to death or disability if diagnosis and treatment are delayed or missed.

The evolution of molecular diagnostic techniques has significantly enhanced the role of genetic testing in GDs diagnosis [2, 6–8]. Recent studies highlight the benefits of genetic testing in clinical settings. For example, a study from China on the largest cohort of neonates with congenital heart defects demonstrated that next-generation sequencing facilitated precise genetic diagnoses, enabling earlier intervention by specialists [9]. A study at Beijing Children’s Hospital [10] revealed that exome sequencing as an initial test for pediatric respiratory diseases had a diagnostic yield of 34.6%, proving its efficacy in rapidly diagnosing and guiding treatment. Additionally, a prospective study [11] showed that using whole-exome sequencing (WES) as a primary test in infants suspected of monogenic disorders could streamline the diagnostic process, offering a higher diagnostic yield than standard approaches. Recent research has also discussed the application of WES in NICU settings and among neonate populations in China from various perspectives [2, 9, 12, 13], including the study of molecular defects in neonates conceived through assisted reproductive technology. Despite these advancements, the application of WES in diagnosing neonatal genetic diseases warrants further exploration. This paper presents a retrospective observational study on a Chinese cohort of neonates, aiming to discuss the significance of WES for patients in the NICU.

## Methods

### Recruitment and data collection

**Fig. 1:**
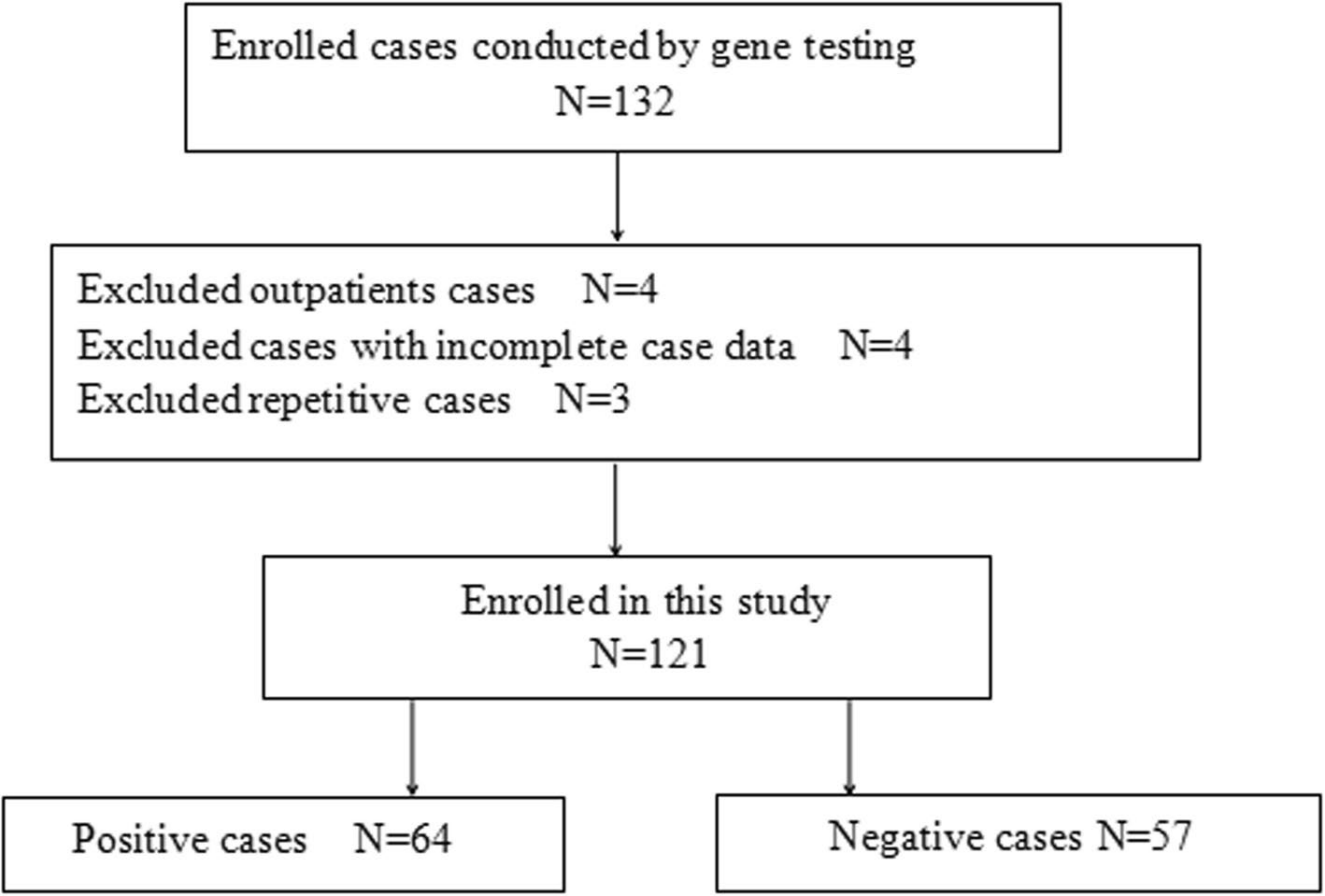
Flow chart of enrolled cases

From January 2018 to April 2021, we analyzed 132 neonates hospitalized in the NICU at Tianjin Children’s Hospital in China, who presented with acute illness and were suspected of having GDs identified through WES. After excluding 11 cases due to clear etiology, incomplete information, or duplicate collections, 121 cases were included in our study, as illustrated in Fig. 1. The Tianjin Children’s Hospital Ethics Committee approved this study, and informed consent was obtained from the guardians or parents. We gathered demographic, prenatal, and intranatal information, along with clinical manifestations, physical examination results, accessory examination outcomes, and family histories of the enrolled patients.

### WES and bioinformatic analysis

Genomic DNA was extracted from the peripheral venous blood of the patients and their parents. WES was conducted by Kingmed Company (Guangzhou, China) and MyGenostics Inc. (Beijing, China), achieving read lengths of 150 bp and an average coverage depth of 100-200X for over 95% of targeted regions. These regions included the coding areas of more than 20,000 genes and the exon–intron boundaries. Exome capture was performed using the xGen Exome Research Panel v2 (Integrated DNA Technologies, US), and sequencing was carried out on the NovaSeq 6000 system (Illumina, US). The raw data were aligned to the human reference genome hg19 using the Burrows-Wheeler Aligner software. Variant annotations were conducted using the ANNOVAR software, updated bi-monthly, with integration from databases [14] such as RefSeq Gene, dbSNP150, ClinVar, HGMD, and allele frequencies from 1000G, ESP6500, and the ExAC database. Copy Number Variants (CNVs) calling was executed using ExomeDepth software [15] with default settings and batch consistency in sequencing and bioinformatics procedures. The common cause of neonatal hypotonia, homozygous deletion of *SMN1*, was analyzed using established protocols [16, 17]. Verification of exome results, when necessary, was done through Sanger sequencing and/or Multiplex Ligation-dependent Probe Amplification (MLPA) using the BigDye™ Terminator v3.1 (Applied Biosystems™, US.) on the ABI3530Dx platform (Applied Biosystems™, US), and standard reagents from MRC-Holland (Netherlands) for MLPA.

### Pathogenicity assessment

The analysis of genetic reports aimed to explore the connection between phenotype and genetic variation. The pathogenicity of variants was classified according to the criteria set by the American College of Medical Genetics and Genomics (ACMG) [18], which includes five levels: pathogenic, likely pathogenic, variant of uncertain significance, likely benign, and benign.

## Results

### Clinical information of neonates in the NICU

This study enrolled 121 neonates, comprising 66 male and 55 female infants, with ages at enrollment ranging from 1 h to 28 days. The majority of the infants were full-term, accounting for 86.8% (105/121), while preterm and extremely preterm infants represented 12.4% (15/121) and 0.8% (1/121), respectively, as detailed in Table 1. The study focused on multiple organ or system involvements, primarily in the metabolic, neurologic, and endocrine systems, with incidence rates of 24% (29/121), 15.7% (19/121), and 12.4% (15/121), respectively, also shown in Table 1. In the metabolic system, the clinical manifestation included poor feeding, vomiting, disturbance of consciousness, metabolic acidosis, hyperammonemia, hyperkalemia, hyperhomocysteinemia, hypotonia, abnormal electroencephalogram, anemia, cardiomyopathy, metabolic alkalosis, hyperphenylalaninemia, lethargy, seizures, coma, poor growth, and intrahepatic cholestasis. The neurologic system's manifestations included conditions such as convulsion, weakness of limbs, hypotonia, loss of tendon reflexes, muscle weakness, inability to suck, axial hypotonia, hearing loss, peculiar hair, weak cry, recurrent bronchopneumonia, swallowing difficulties, respiratory distress, while the endocrine system showed signs such as electrolyte disorders, dark areola, vomiting, poor feeding, and malnutrition were observed in the endocrine system. Additional details on organ involvement are provided in Supplemental Table 1.

**Table 1:** Clinical characteristics of neonates

Total number of neonates	*N* = 121
Gender	n (%)
Male	66 (54.5)
Female	55 (45.5)
Gestational age	n (%)
Post-term(≥ 42 weeks)	0 (0.0)
Term(37-42 weeks)	105 (86.8)
Preterm(28-37 weeks)	15 (12.4)
Extremely preterm(< 28 weeks)	1 (0.8)
Organ system involvement	n (%)
Resparitory/Pulmonary	4 (3.3)
Cardiovascular	4 (3.3)
Gastrointestinal	9 (7.4)
Neurologic	19 (15.7)
Hematologic	5 (4.1)
Endocrine	15 (12.4)
Renal	2 (1.7)
Immunologic	7 (5.8)
Metabolic	29 (24.0)
Musculoskeletal	4 (3.3)
Dermatologic	4 (3.3)
Audiologic	3 (2.5)
Craniofacial	3 (2.5)
Ophthalmologic	1 ( 0.8)
multiple organs	12 (9.9)

### Variants information of neonates in the NICU

**Fig. 2:**
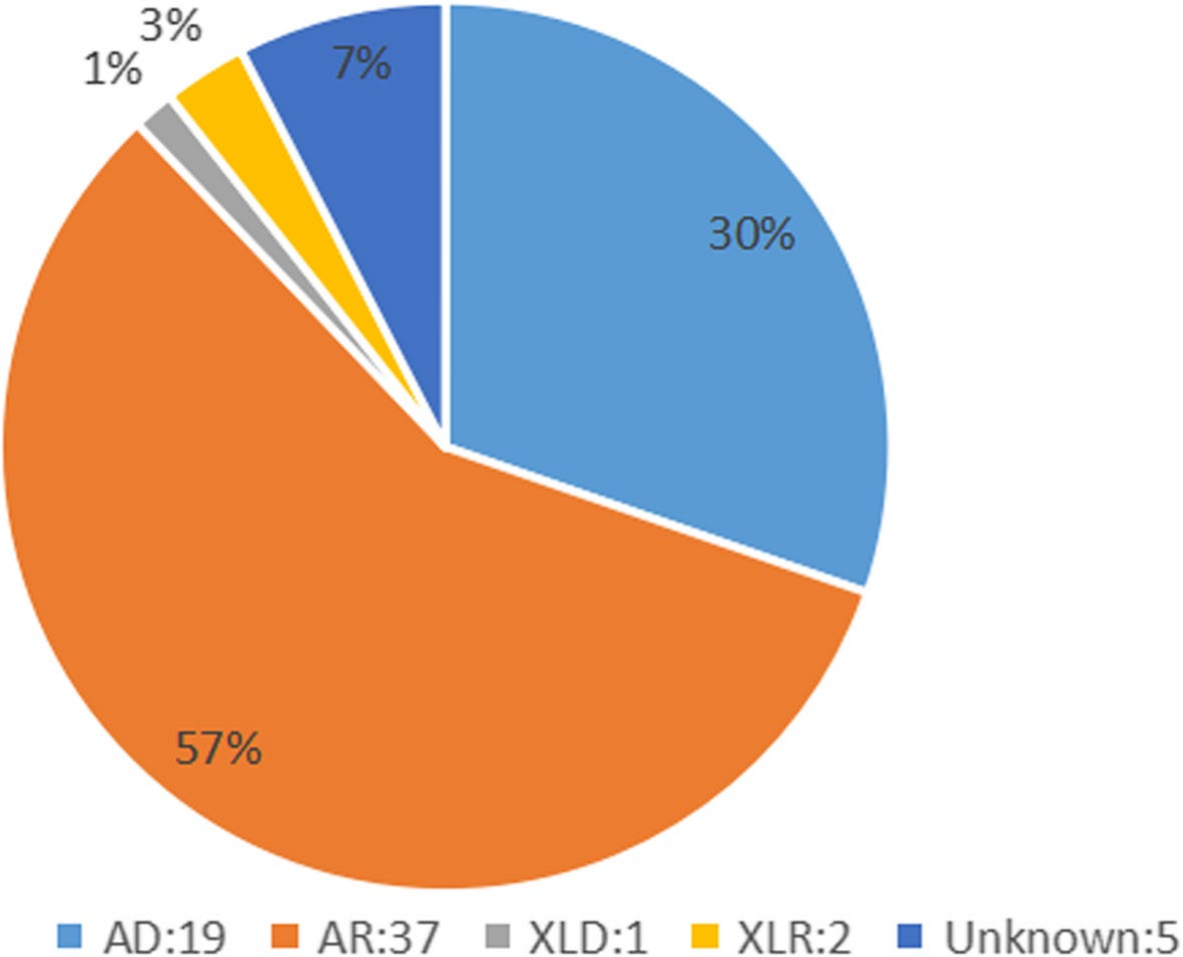
Proportion of inheritance patterns. Abbreviations: AD, autosomal recessive; AR, autosomal dominant; XLD, X-linked dominant; XLR, X-linked recessive

The overall detection rate of pathogenic or likely pathogenic genomic variants via WES was 52.9% (64/121). We identified 84 genomic variants in 64 neonates, comprising 13 copy number variations (CNVs) and 71 single-nucleotide variants (SNVs), detailed in Table 2. The CNVs mostly involved deletions and duplications, including cases of spinal muscular atrophy and global developmental delay or multiple malformations due to various CNVs. SNVs were primarily missense, nonsense, frameshift, and splicing variations. De novo variations numbered 16 in this cohort, as shown in Table 2. Parental samples were collected simultaneously with the offspring samples for verification by Sanger sequencing upon detection of variant genes. The inheritance patterns are detailed in Fig. 2, with autosomal recessive being the most common (57.8%, 37/64), followed by autosomal dominant (29.7%, 19/64). There was one case of X-linked dominant inheritance, two of X-linked recessive, and five of unknown inheritance patterns. In 4 cases of CNVs, testing at the corresponding loci was recommended for both parents, though it was not performed due to a lack of parental permission. In one case, patient 34 exhibited only one heterozygous pathogenic variant in the *FGA* gene, suggesting the possibility of undetected mutations by WES.

**Table 2:** Details of genetic findings in 64 neonates of the current cohort

ID	Gene	Gene variant(s)	Reference sequence	Zygosity	Inherited pattern	The source of variation	Variation type	Variation classification	Evidence	Diagnosis (OMIM ID)
Patient 1	*EXT2*	c.514C > T/p.(Gln172*)	NM_207122.1	Het	AD	Father	Nonsense variation	P	^PM2_Supporting, PM3_Strong, PVS1^	^#^Exostoses, multiple, type 2 (133,701)
Patient 2	*CYP21A2*	c.293-13C > G/ p.? c.1069C > T/p.(Arg357Trp)	NM_000500.7	Het Het	AR AR	Mother Father	Splicing variation Missense variation	LP P	PM2_Supporting, PP4, PM3_VeryStrong, PS3_Supporting, BP4 _ Moderate /PM2_Supporting, PP4, PM3_Strong, PS3_Moderate, PP3_Supporting, PM1	Adrenal hyperplasia, congenital, due to 21-hydroxylase deficiency (201,910)
Patient 3	*UGT1A1*	c.1091C > T/p.(Pro364Leu)	NM_000463.2	Het	AD	Father	Missense variation	LP	^PP4, PS3_Supporting, PP3_ Moderate, PM1^	^#^Hyperbilirubinemia, familial transient neonatal (237,900)
Patient 4	*SMN1*	seq[GRCh37]del(5)(q13.2)chr5:g.21126-21236del seq[GRCh37]del(5)(q13.2)chr5:g.27001-27054del	NM_000344.3	Homo	AR	Parent	Deletion variation	P	1A(0), 2A(1), 3A(0), 4 M(0.3)	Spinal muscular atrophy-1 (253,300)
Patient 5	*MMACHC*	c.80A > G/p.(Gln27Arg) c.81 + 1G > A/p.?	NM_015506.2	Het Het	AR AR	Mother Father	Missense variation Splicing variation	LP LP	PM2_Supporting, PP4, PM3_Strong, PM1/PM2_Supporting, PP4, PM3_Strong	Methylmalonic aciduria and homocystinuria, cblC type (277,400)
Patient 6	*STS*	Chrxp22.31 del (including STS gene)	-	Hemi	XLR	De novo	Deletion variation	P	^1A(0), 2A(1), 3A(0), 4A(0.9)^	^#^Ichthyosis, X-linked (308,100)
Patient 7	*ATP6V0A2*	c.117 + 5G > T/p.?	NM_012463.3	Homo	AR	Parent	Splicing variation	LP	^PM2_Supporting, PVS1^	^#^Wrinkly skin syndrome (278,250)
Patient 8	*MMACHC*	c.80A > G/p.(Gln27Arg) c.217C > T/p.(Arg73*)	NM_015506.2	Het Het	AR AR	Mother Father	Missense variation Nonsense variation	LP P	PM2_Supporting, PP4, PM3_Strong, PM1/PM2_Supporting, PP4, PM3_Strong, PVS1	Methylmalonic aciduria and homocystinuria, cblC type (277,400)
Patient 9	*SCN2A*	c.781G > A /p.(Val261Met)	NM_021007.2	Het	AD	Father	Missense variation	P	PM2_Supporting, PS4, PS3 _Supporting, PP3_Strong, PM1	Seizures, benign familial infantile, 3, 607,745
Patient 10	*KCNQ2*	seq[GRCh37]del(20)(q13.33)chr20:g.-del	-	Het	AD	De novo	Deletion variation	P	1A(0), 2A(1), 3A(0)	Seizures, benign neonatal, 1 (121,200)
Patient 11	*SPTB*	c.3737delA/p.(Lys1246fs)	NM_000347.5	Het	AD	Father	Deletion variation	P	^PM2_Supporting, PP4, PVS1^	^#^Spherocytosis, type 2 (616,649)
Patient 12	*SMN1*	seq[GRCh37]del(5)(q13.2)chr5:g.21126-21236del seq[GRCh37]del(5)(q13.2)chr5:g.27001-27054del	NM_000344.3	Homo	AR	-	Deletion variation	P	1A(0), 2A(1), 3A(0), 4 M(0.3)	Spinal muscular atrophy-1 (253,300)
Patient 13	*KCNQ2*	c.797A > G/p.(Asp266Gly)	NM_172107.2	Het	AD	De novo	Missense variation	P	PM2_Supporting, PS4, PS2 _Supporting, PM5_supporting, PP3 _Moderate, PM1	Seizures, benign neonatal, 1 (121,200)
Patient 14	*MMUT*	c.1677-1G > C/p.?	NM_000255.3	Homo	AR	Parent	Splicing variation	P	PM2_Supporting, PM3_strong, PVS1	Methylmalonic aciduria, mut(0) type (251,000)
Patient 15	*RUNX2*	c.539C > A/p.(Ala180Glu)	NM_001024630.3	Het	AD	De novo	Missense variation	P	^PM2_supporting, PS2_moderate, PS3_supporting, PP3 _strong, PM1^	^#^Cleidocranial dysplasia (119,600)
Patient 16	*KCNQ2*	c.629G > A/p.(Arg210His)	NM_172107.2	Het	AD	De novo	Missense variation	P	PM2_supporting, PS4, PM5_supporting, PP3 _moderate, PM1	Seizures, benign neonatal, 1 (121,200)
Patient 17	*PKHD1*	c.2264C > T/p.(Pro755Leu) c.1969C > T/p.(Gln657*)	NM_138694.3	Het Het	AR AR	Mother Father	Missense variation Nonsense variation	LP LP	^PM2_supporting, PM3_strong, PP3 _moderate, PM1/PM2_supporting, PVS1^	^#^Polycystic kidney disease 4, with or without hepatic disease (263,200)
Patient 18	*GJB2*	c.35G > A p.(Gly12Asp)	NM_004004.5	Het	AD	Mother	Missense variation	LP	^PM2_Supporting, PM5_supporting, PP3_moderate, PM1^	^#^Deafness, autosomal dominant 3A (601,544)
Patient 19	*ASS1*	c.380G > T/p.(Arg127Leu)	NM_000050.4	Homo	AR	Parent	Missense variation	LP	^PM2_Supporting, PP4, PP3_Strong, PM1^	^#^Citrullinemia (215,700)
Patient 20	*VPS13B SLC26A4*	c.6940 + 1G > T/p.? c.919-2A > G/p.?	NM_017890.4 NM_000441.1	Homo Homo	AR AR	Parent Parent	Splicing variation Splicing variation	P P	PM2_Supporting, PM3_strong, PVS1/PM3_strong, PVS1	Cohen syndrome (216,550) ^#^Deafness, autosomal recessive 4, with enlarged vestibular aqueduct (600,791)
Patient 21	-	seq[GRCh37]dup(5)(p15.33p15.2)chr5:g.140413-14769310dup seq[GRCh37]del(18)(q12.31q23)chr18:g.55711883-78005241del	-	-	-	-	Duplication variation Deletion variation	P P	1A(0), 2H(0), 3C(0.9), 4 M(0.3)/1A(0), 2H(0.15), 3C(0.9), 4 M(0.3)	
Patient 22	*SMN1*	seq[GRCh37]del(5)(q13.2)chr5:g.21126-21236del seq[GRCh37]del(5)(q13.2)chr5:g.27001-27054del	NM_000344.3	Homo	AR	Parent	Deletion variation	P	1A(0), 2A(1), 3A(0), 4 M(0.3)	Spinal muscular atrophy-1 (253,300)
Patient 23	*CYP21A2*	c.293-13C > G/p.? Exon1/3/4/6/7 deletion	-	Het Het	AR AR	Mother De novo	Splicing variation Deletion variation	LP VUS	PM2_Supporting, PP4, PM3_VeryStrong, PS3_Supporting,BP4 _ Moderate/1A(0), 3A(0)	Adrenal hyperplasia, congenital, due to 21-hydroxylase deficiency (201,910)
Patient 24	*MMACHC*	c.609G > A/p.(Trp203*) c.567dupT/p.(Ile190fs)	NM_015506.2	Het Het	AR AR	Father Mother	Nonsense variation Frameshift variation	P P	PM2_Supporting, PM3_Strong, PVS1/PM2_Supporting, PM3_Strong, PVS1	Methylmalonic aciduria and homocystinuria, cblC type (277,400)
Patient 25	*ABCA3*	c.115C > G/p.(Leu39Val) c.277G > A/p.(Val93Ile)	NM_001089.2	Het Het	AR AR	Father Mother	Missense variation Missense variation	LP VUS	PP4, PM3_strong, PP3_moderate, PM1/PP4, PP3, PM1	^#^Surfactant metabolism dysfunction, pulmonary, 3 (610,921)
Patient 26	*CASK*	c.764G > A/p.(Arg255His)	NM_003688.3	Hemi	XLD	De novo	Missense variation	LP	^PM2_Supporting, PS4, PM5_supporting, PM1^	^#^Intellectual developmental disorder and microcephaly with pontine and cerebellar hypoplasia (300,749)
Patient 27	*SMN1*	seq[GRCh37]del(5)(q13.2)chr5:g.21126-21236del seq[GRCh37]del(5)(q13.2)chr5:g.27001-27054del	NM_000344.3	Homo	AR	Parent	Deletion variation	P	1A(0), 2A(1), 3A(0), 4 M(0.3)	Spinal muscular atrophy-1 (253,300)
Patient 28	*GAA*	c.859-2A > T/p.? c.1861 T > G/p.(Trp621Gly)	NM_000152.3	Het Het	AR AR	Mother Father	Splicing variation Missense variation	P VUS	^PM2_Supporting, PM3_Strong, PVS1/PM2_Supporting, PP3_moderate, PM1^	^#^Glycogen storage disease II (232,300)
Patient 29	*ANK1*	c.3365delT/p.(Leu1122Arg)	NM_000037.3	Het	AD	Mother	Frameshift variation	LP	^PM2_Supporting, PVS1^	^#^Spherocytosis, type 1 (182,900)
Patient 30	*ZMPSTE24*	c.743C > T/p.(Pro248Leu) seq[GRCh37]del(1)(p34.2)chr1:g.40747005-41013142del	NM_005857.4	Het Het	AR AR	Father -	Missense variation Deletion variation	P VUS	^PM2_Supporting, PP4, PM3_Strong, PP3_moderate, PM1/1A(0), 3A(0)^	^#^Mandibuloacral dysplasia with type B lipodystrophy, 608,612 ^#^Restrictive dermopathy 1 (275,210)
Patient 31	*PCCA*	c.2002G > A/p.(Gly668Arg)	NM_000282.3	Homo	AR	Parent	Missense variation	P	PM2_Supporting, PM3_Strong, PP3_strong, PM1	Propionicacidemia (606,054)
Patient 32	*PCCA*	c.2002G > A/p.(Gly668Arg)	NM_000282.3	Homo	AR	Parent	Missense variation	P	PM2_Supporting, PM3_Strong, PP3_strong, PM1	Propionicacidemia, (606,054)
Patient 33	*ATP7A*	c.2383C > T/p.(Arg795*)	NM_000052.6	Homo	XLR	Mother	Nonsense variation	P	^PM2_Supporting, PS4, PVS1^	^#^Menkes disease (309,400)
Patient 34	*FGA*	c.104G > A/ p.(Arg35His)	NM_021871.2	Het	-	Mother	Missense variation	P	^PM2−supporting, PS4, PM5_Strong, PP3^	^#^Afibrinogenemia, congenital (202,400)
Patient 35	-	seq[GRCh37]del(2)(q37.2q37.3)chr2:g.236403321-242841491del seq[GRCh37]dup(22)(q11.21)chr22:g.20920744-21154064dup seq[GRCh37]dup(18)(q23)chr18:g.74074443-78005241dup	-	-	-	-	Deletion variation Duplication variation Duplication variation	P VUS VUS	1A(0),2A(1),2H(0.15),3C(0.9),4 M(0.3)/1A(0),3A(0)/1A(0),3A(0),4 M(0.3)	
Patient 36	*CPS1*	c.2339G > A/p.(Arg780His) c.3520C > T/p.(Arg1174*)	NM_001875.4	Het Het	AR AR	Father Mother	Missense variation Nonsense variation	LP P	^PM2_Supporting,PM3_Strong,PM5,PP3_Moderate/PVS1,PM2_Supporting,PM3_Strong^	^#^Carbamoylphosphate synthetase I deficiency (237,300)
Patient 37	*SPTB*	c.4735C > T/p.(Arg1579*)	NM_000347.5	Het	AD	De novo	Nonsense variation	P	^PVS1,PS4,PM2_Supporting,PP4^	^#^Spherocytosis, type 2 (616,649)
Patient 38	*PAX2*	c.76dupG/p.(Val26fs)	NM_003987.4	Het	AD	De novo	Frameshift variation	P	^PVS1,PS4^	^#^Papillorenal syndrome (120,330)
Patient 39	*CYP21A2*	c.293-13C > G/p.?	NM_000500.7	Homo	AR	Parent	Splicing variation	P	PS3,PM3_VeryStrong,PP4	Adrenal hyperplasia, congenital, due to 21-hydroxylase deficiency (201,910)
Patient 40	*STXBP1*	c.578 + 1G > T/p.?	NM_003165.3	Het	AD	De novo	Splicing variation	P	PVS1,PM2_supporting, PM3_Strong	Developmental and epileptic encephalopathy 4 (612,164)
Patient 41	*NF1*	c.3044 T > C/p.(Leu1015Pro)	NM_000267.3	Het	AD	De novo	Missense variation	P	^PM2_Supporting,PS4,PM1,PM5,PP3^	^#^Neurofibromatosis, type 1 (162,200)
Patient 42	*MMUT*	c.2131G > T/p.(Glu711*) c.1889G > A/p.(Gly630Glu)	NM_000255.3	Het Het	AR AR	De novo Father	Nonsense variation Missense variation	LP P	PVS1_Moderate,PM2_supporting,PM3_Strong/PM1,PM2_Supporting,PM3_Strong,PM5,PP3_Strong	Methylmalonic aciduria, mut(0) type (251,000)
Patient 43	*SCN2A*	c.2657 T > C/p.(Leu886Ser)	NM_021007.2	Het	AD	De novo	Missense variation	P	PS4,PM1,PM2_Supporting,PP3_Strong	Developmental and epileptic encephalopathy 11 (613,721)
Patient 44	*NIPBL*	c.5366G > T/p.(Arg1789Leu)	NM_133433.3	Het	AD	De novo	Missense variation	P	^PS4,PM1,PM2_Supporting,PM5,PP3_Moderate^	^#^Cornelia de Lange syndrome 1 (122,470)
Patient 45	*TTN*	c.89675delA p.(Lys29892fs)	NM_133378.4	Het	AD	-	Frameshift variation	LP	^PM2_Supporting, PVS1^	^#^Myopathy, myofibrillar, 9, with early respiratory failure (603,689)
Patient 46	-	seq[GRCh37]dup(1)(q42.13q44)chr1:g.228969151-249224684dup seq[GRCh37]del(9)(p24.3p23)chr9:g.208455-10287179del	-	-	-	-	Duplication variation Deletion variation	P P	1A(0), 2G(0), 2H(0), 3C(0.9), 4 M(0.3) /1A(0),2A(1), 2H(0.15), 3B((0.45), 4 M(0.3)	
Patient 47	*PTS*	c.286G > A/p.(Asp96Asn) c.317C > T/p.(Thr106Met)	NM_000317.2	Het Het	AR AR	Father Mother	Missense variation Missense variation	P LP	^PM2_Supporting,PM3_VeryStrong,PP3_Moderate/PM2_Supporting,PM3_Strong,PP3_Strong^	^#^Hyperphenylalaninemia, BH4-deficient, A (261,640)
Patient 48	*CYP21A2*	c.293-13C > G/p.? Exon1/3 del	NM_000500.7	Het Het	AR AR	Mother -	Splicing variation Deletion variation	P LP	PS3,PM3_VeryStrong,PP4/ PM2_Supporting,PVS1	Adrenal hyperplasia, congenital, due to 21-hydroxylase deficiency (201,910)
Patient 49	*MMACHC*	c.80A > G/p.(Gln27Arg) c.658_660del/p.(Lys220del)	NM_015506.2	Het Het	AR AR	Father Mother	Missense variation Deletion variation	LP LP	PM2_Supporting,PM3_VeryStrong/PM2_Supporting,PM3_VeryStrong	Methylmalonic aciduria and homocystinuria, cblC type (277,400)
Patient 50	*PLOD1*	c.1095C > T/p.(Gly365 =)	NM_000302.3	Homo	AR	Parent	Same sense variation	P	^PM2_Supporting,PM3_Strong,PP3,PS3^	^#^Ehlers-Danlos syndrome, kyphoscoliotic type, 1 (225,400)
Patient 51	*VPS13B*	c.4213delG/p.(E1405Kfs*4) c.10244C > T/p.(T3415I)	NM_152564 NM_017890	Het Het	AR AR	Mother Father	Frameshift variation Missense variation	LP VUS	PVS1,PM2_Supporting/PM2_Supporting,PM3,	Cohen syndrome (216,550)
Patient 52	*IGHMBP2*	c.1813C > T/p.(Arg605*) c.905_912 + 84del/p.(Asp302fs)	NM_002180.2	Het Het	AR AR	Mother Father	Nonsense variation Frameshift variation	P VUS	^PVS1,PM2_Supporting,PM3_Strong/PVS1_Moderate,PM2_Supporting^	^#^Neuronopathy, distal hereditary motor, autosomal recessive 1 (604,320)
Patient 53	*SLC26A2*	c.1020_1022delTGT/p.(V341del) c.800C > T/p.(S267F)	NM_000112	Het Het	AR AR	Mother Father	Deletion variation Missense variation	LP VUS	^PM2_Supporting,PM3_Strong,PM4/PM2_Supporting,PP3_Strong^	^#^Atelosteogenesis, type II (256,050) ^#^Achondrogenesis Ib (600,972)
Patient 54	*KCNQ2*	c.941C > G/p.(Ser314Cys)	NM_172107.3	Het	AD	De novo	Missense variation	LP	PM1,PM2_Supporting,PM5,PP3_Strong	Developmental and epileptic encephalopathy 7 (613,720)
Patient 55	*HADH*	c.493C > T/p.(R165*) c.89 T > A/p.(V30E)	NM_005327	Het Het	AR AR	Father Mother	Nonsense variation Missense variation	LP LP	^PVS1,PM2_Supporting/PM2_Supporting,PM3_Strong,PP3_Moderate^	^#^3-hydroxyacyl-CoA dehydrogenase deficiency (231,530), ^#^Hyperinsulinemic hypoglycemia, familial, 4 (609,975)
Patient 56	*MMACHC*	c.315C > G/p.(Y105*) c.481C > T/p.(R161*)	NM_015506	Het Het	AR AR	Mother Father	Nonsense variation Nonsense variation	P P	PVS1,PM2_Supporting,PM3_Strong/PVS1,PM2_Supporting,PM3_Strong	Methylmalonic aciduria and homocystinuria, cblC type (277,400)
Patient 57	*MMACHC*	c.445_446delTG/p.(Cys149fs*32) c.658_660delAAG/p.(Lys220del)	NM_015506	Het Het	AR AR	Father Mother	Frameshift variation Deletion variation	P P	PVS1,PM2_Supporting,PM3_Strong/PS3,PM3_VeryStrong,PM4	Methylmalonic aciduria and homocystinuria, cblC type (277,400)
Patient 58	*BCKDHB*	c.550delT/p.(Ser184fs) c.508C > G/p.(Arg170Gly)	NM_183050.3	Het Het	AR AR	Mother Father	Frameshift variation Missense variation	P P	^PVS1,PM2_Supporting,PM3_Strong/PM1,PM2_Supporting,PM3_Strong,PM5,PP3_Moderate^	^#^Maple syrup urine disease, type Ib (620,698)
Patient 59	-	seq[GRCh37]del(12)(p13.33)chr12:g.173787-3077219del seq[GRCh37]dup(X)(q27.3q28)chrX:g.145751423-155233731dup	-	-	-	-	Deletion variation Duplication variation	VUS P	1A(0),2H(0.15),3A(0),4 M(0.3)/1A(0),2A(1),2H(0.15),3C(0.9),4 M(0.3)	
Patient 60	*PAH*	c.478C > T/p.(Q160*) c.688G > A/p.(V230I)	NM_000277	Het Het	AR AR	Mother Father	Nonsense variation Missense variation	P P	PVS1,PM2_Supporting,PM3_Strong/PM2_Supporting,PM3_VeryStrong,PM5,PP4_Moderate	Phenylketonuria (261,600)
Patient 61	*SLC26A4*	c.919-2A > G/(p.splicing)	NM_000441	Homo	AR	Parent	Splicing variation	P	^PVS1, PM3_VeryStrong, PP1_Strong^	^#^Pendred syndrome (274,600)
Patient 62	*PKHD1*	c.4274 T > G/p.(L1425R) c.2507 T > C/p.(V836A)	NM_138694	Het Het	AR AR	Mother Parent	Missense variation Missense variation	LP P	^PM2_Supporting;PM3_Strong;PP3_Moderate/PP3_Moderate;PM3_VeryStrong;PM2_Supporting^	^#^Polycystic kidney disease 4, with or without hepatic disease (263,200)
Patient 63	*SLC25A13*	c.852_855delTATG/p.M285Pfs*2 IVS4ins6kb	NM_014251	Het Het	AR AR	Father -	Frameshift variation Insert variation	P LP	PVS1,PM3_VeryStrong/PVS1, PM2_Supporting	^#^Citrullinemia, type II, neonatal-onset (605,814)
Patient 64	*CHD7*	c.5569delT/p.(Y1857Ifs*12)	NM_017780	Het	AD	De novo	Frameshift variation	LP	^PVS1,PM2_Supporting^	^#^CHARGE syndrome (214,800)

*Abbreviations*: *AD* autosomal dominant, *AR* autosomal recessive, *Hemi* hemizygous, *Het* heterozygous, *Homo* homozygous, *LP* likely pathogenic, *P* pathogenic, *VUS* variant of uncertain significance, *XL* X-linked, *XLD* X-linked dominant, *XLR* X-linked recessive,—unknown/absent

### Genetic disorders diagnosed by WES

**Fig. 3:**
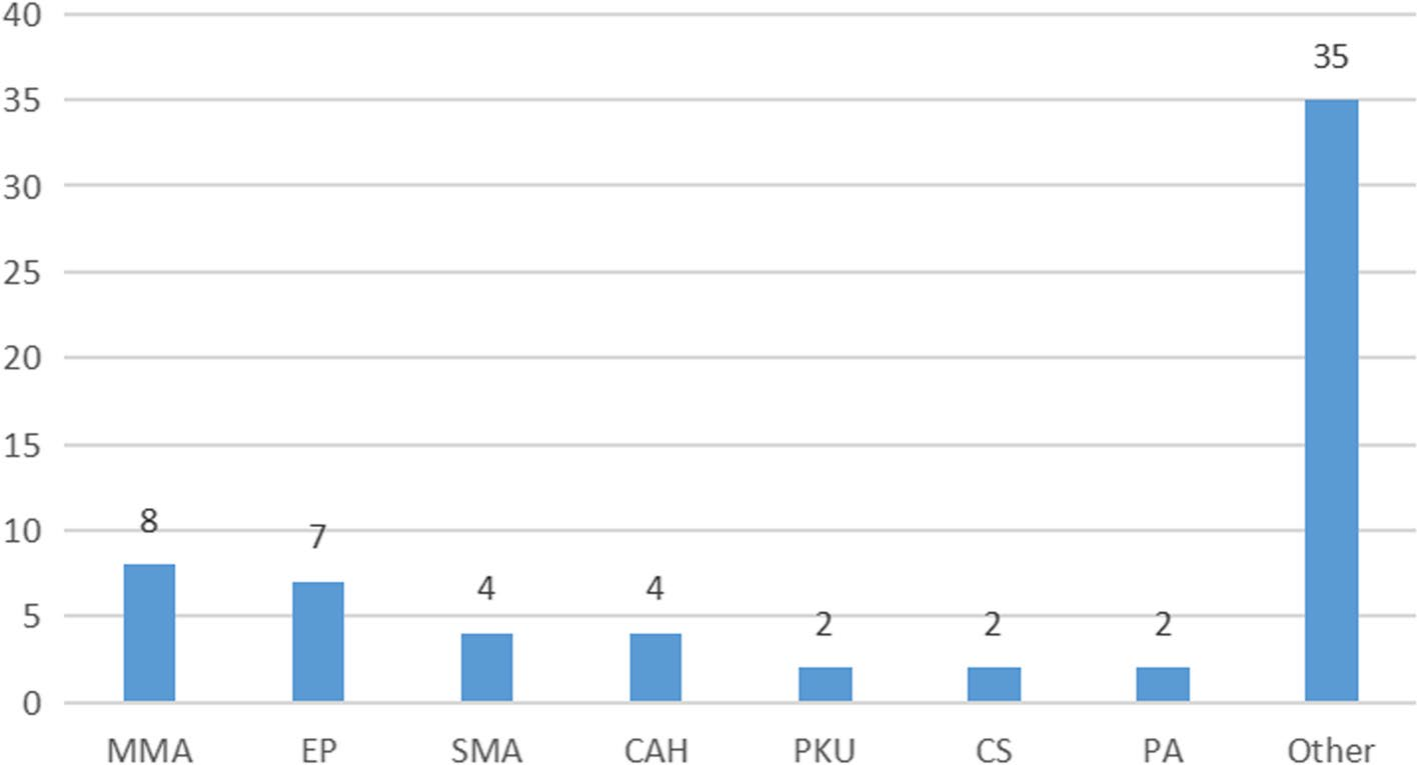
Distribution of diagnosed diseases. Abbreviations: CAH, congenital adrenal hyperplasia; CS, Cohen syndrome; Ep, epilepsy; MMA, methylmalonic acidemia; PA, propionic acidemia; PKU, phenylketonuria; SMA, spinal muscularatrophy; Note: the details of “Other” category shown in Table 2 with superscripted “#”

WES identified 40 diseases within the study cohort. The most prevalent disorders were methylmalonic acidemia (MMA) (12.5%, 8/64), epilepsy (10.9%, 7/64), spinal muscular atrophy (6.25%, 4/64), and congenital adrenal hyperplasia (6.25%, 4/64), as shown in Fig. 3. Notably, the audiologic system showed a 100% positive rate (3/3), followed by the neurologic system at 68.4% (13/19) in this cohort.

## Discussion

WES has become a primary clinical diagnostic tool for children suffering from developmental delays, intellectual disabilities, respiratory disease [10, 19], and more. Recent discussions have highlighted its use in NICU settings and among neonatal populations in China, viewing it from various perspectives [2, 9, 12, 13]. Despite these discussions, the potential of WES in diagnosing neonatal GDs remains underexplored. In our study, WES was conducted on 121 infants in the NICU at Tianjin Children's Hospital of China, yielding a diagnostic rate of 52.9% (64/121). This rate surpasses the 37.9% diagnostic yield of a similar study in the USA [20] and significantly exceeds the 12.3% yield reported in Lin Yang's studies in China [13]. For the discrepancy between this study and two other studies, we analyzed potential reasons based on differences in patient selection and cohort size. Our cohort, selected with stricter inclusion criteria, demonstrated a higher diagnostic yield. We focused on critically ill patients in the NICU, with strong indications for GDs assessed by experienced experts, such as evidence of metabolic disorders identified by mass spectrometry. Additionally, this was a single-center study conducted at Tianjin Children’s Hospital, which also accepted critically ill patients from surrounding districts. The cohort size was relatively small, with fewer patients than in the study by Lin Yang et al. [13], which included a cohort of 2,303 neonates in China. In our cohort, a significant proportion of genetically diagnosed patients had metabolic disorders, such as methylmalonic acidemia (MMA), hyperphenylalaninemia, and congenital adrenal hyperplasia. Despite most patients undergoing newborn screening techniques, some reports were negative or showed suspected positive results. In these cases, WES was performed to confirm diagnoses. Among the positive cases, epilepsy and MMA were the most frequently identified diseases, accounting for 10.9% (7/64) and 12.5% (8/64) of cases, respectively.

MMA arising from either a deficiency in methylmalonyl-CoA or abnormal cobalamin metabolism, is a rare, inherited metabolic disorder, primarily passed down through autosomal recessive inheritance. It stands as the most common form of organic acidemia [21]. The disease's genetic underpinnings include mutations in several genes, such as *MMACHC*, *MMADHC*, and *MMUT*, with the prevalence of these mutations varying across different countries and populations [22–24]. In China, for instance, the most common mutations in children with MMA are c.609G > A, c.658_660delAAG, and c.80A > G in the *MMACHC* gene, occurring at frequencies of 34.09%, 13.64%, and 13.64%, respectively [22]. Our study of 8 MMA cases revealed 12 mutations across two genes (*MMACHC* and *MMUT*), which included a novel mutation in *MMUT* c.2131G > T/p.(Glu711*) and eleven inherited mutations. We identified nine gene variations in the *MMACHC* gene and three mutations in the *MMUT* gene. The frequency of c.658_660delAAG and c.80A > G mutations was 16.7% (2/12) and 25.0% (3/12), respectively, aligning with findings from previous studies in China [22, 23]. Among eight cases, one was homozygous, while the others were compound heterozygous. All eight MMA patients exhibited an autosomal recessive inheritance pattern. The manifestations of MMA can be nonspecific and vary among patients, especially in newborns and young infants. Previous research has shown that the clinical course of MMA can progress rapidly in neonates, sometimes resulting in death [25, 26] if not treated promptly. Fortunately, MMA is a treatable genetic disorder for most patients [27]. Our team [28] reported a case of a neonate with MMA metabolic decompensation (severe metabolic acidosis and hyperammonemia (> 1,000 µg/dl) who was successfully treated with automatic peripheral arteriovenous exchange transfusion and L-carnitine. The diagnosis was confirmed by WES, leading to a decrease in serum ammonia levels and an improvement in the child’s clinical status. Therefore, WES is crucial for diagnosing this disease, enabling timely treatment and improving the prognosis for NICU patients.

Neonatal seizures are a common manifestation of neurological dysfunction, with an incidence of about 1–5 per 1,000 births [29, 30]. Despite a decrease in mortality from 40 to 20%, the prognosis for neurodevelopmental outcomes, such as cerebral palsy, intellectual disability, and secondary epilepsy, has not significantly improved [30]. Therefore, identifying the cause of neonatal seizures and initiating timely medical treatment is crucial for managing these conditions. The causes of neonatal seizures are diverse, including acute symptomatic seizures, electrolyte imbalances, and cerebral deformity, and so on [30, 31]. Recent advancements in molecular diagnostic technologies, such as WES, have increased the detection rate of genetic disorders causing neonatal seizures [32]. In this study, we identified seven neonatal patients with seizures, uncovering seven mutations in three pathogenic genes. These included five missense mutations, one deletion mutation, and one splicing mutation, comprising one inherited and six de novo variations. For instance, in patient 9, the *SCN2A* c.781G > A /p.(Val261Met) mutation was identified, leading to a diagnosis of benign familial neonatal convulsions, a form of epilepsy with a favorable prognosis. Thus, WES is valuable for pinpointing genetic causes and guiding precise treatments in NICUs.

In another case, patient 28, a female term infant, was admitted to the NICU of Tianjin Children’s Hospital at 25 days old due to jaundice and elevated liver enzymes. The patient, born to a 32-year-old mother, had a normal birth history. Apart from jaundice, the physical examination was unremarkable. Lab tests revealed elevated creatine kinase and glutamic-pyruvic transaminase levels, and an ultrasound cardiogram showed ventricular hypertrophy, suggesting neonatal jaundice, liver dysfunction, and potential hypertrophic cardiomyopathy. Given the unclear etiology, she opted for symptomatic treatment and underwent WES detection. Genetic testing revealed two heterozygous mutations in the *GAA* gene on chromosome 17q25: c.859-2A > T (p.?) and c.1861 T > G (p.Trp621Gly), confirming the diagnosis of glycogen storage disease type II, also known as Pompe disease. This diagnosis was significantly different from the initial assumption. Pompe disease, a rare autosomal recessive disorder caused by mutations in the *GAA* gene, leads to a chronic and progressive pathology, predominantly featuring limb-girdle muscle weakness and respiratory failure [33]. Early diagnosis is crucial to mitigate or prevent the irreversible organ damage that progresses with Pompe disease [34]. However, our patient presented without the typical clinical phenotype at admission, posing a diagnostic challenge for clinicians. Thus, WES served as a critical diagnostic tool for patients with unexplained symptoms, ranging from isolated hyper-CKemia to varying degrees of muscular impairment. This case underscores the importance of WES in diagnosing patients with suspected genetic disorders in the NICU, particularly when clinical phenotypes vary widely.

WES analysis presented a negative diagnostic yield of 47.1% (57/121) within this cohort, potentially constrained by the limitations inherent to WES. While WES can detect a wide array of variants, it has a restricted capability in identifying non-coding region variants, abnormal genomic structures, and genomic methylation [35, 36]. However, WES offers significant advantages. Firstly, it covers a broad range of detection and has become more affordable, making it accessible to most parents. Secondly, WES is invaluable for precise diagnosis and treatment strategies, potentially increasing the number of diagnosed infants in NICUs with GDs, thereby reducing infant mortality and morbidity through early neonatal diagnosis. Lastly, WES plays a crucial role in genetic counseling for parents of infants with GDs, enabling informed reproductive decisions. As a vital supplement to standard diagnostics, WES is indispensable for diagnosing GDs in NICU patients.

## Conclusions

In conclusion, our findings underscore the critical role of WES in uncovering the etiology, offering targeted therapy, and enhancing the prognosis for patients with suspected GDs in NICUs, especially when diagnoses are complicated by diverse clinical phenotypes. Reflecting on this study and the evidence gathered from this cohort, we advocate for WES as the primary testing approach for suspected GDs cases in NICUs. This recommendation aligns with the evolving trend towards precision medicine, highlighting WES's clinical utility and its importance to patients and their families in cases lacking a clear diagnosis.

### Supplementary Information

Supplementary Material 1.

## Data Availability

All data generated or analyzed in this study are included in this published article.

## Abbreviations

GDs: Genetic disorders
NICU: Neonatal intensive care unit
WES: Whole-exome sequencing
CNVs: Copy number variations
MLPA: Multiplex ligation-dependent probe amplification
SNVs: Single-nucleotide variants
MMA: Methylmalonic acidemia
