# Supplementary material for: Whole-exome sequencing as the first-tier test for patients in neonatal intensive care unit: a Chinese single-center study
Source: BMC Pediatr. 2024 May 22;24:351. doi: 10.1186/s12887-024-04820-0 (PMC11110365; doi:10.1186/s12887-024-04820-0)
Supplement: Supplementary file 1 — Supplementary Material 1. [file 12887_2024_4820_MOESM1_ESM.docx]

Supplemental Table 1. The detailed clinical manifestations

| System involvement | Clinical phenotypes |
| --- | --- |
| Resparitory/Pulmonary | full-term infant with neonatal respiratory distress |
| Gastrointestinal | hyperbilirubinemia, jaundice |
| Neurologic | weakness of limbs, hypotonia, loss of tendon reflexes, convulsion, muscle weakness, inability to suck, axial hypotonia, hearing loss, peculiar hair, weak cry, recurrent bronchopneumonia, swallowing difficulties, respiratory distress |
| Hematologic | anemia, jaundice, splenomegaly, spherocytes on the peripheral blood smear, hypofibrinogenemia |
| Endocrine | electrolyte disorders, hyponatremia, hyperkalemia, dark areola, vomiting, poor feeding, malnutrition |
| Renal | hypokalemia, hypochloridemia, metabolic alkalosis, polycystic kidney, cystic nodules of the liver, respiratory failure, atrial septal defect |
| Immunologic | thrombocytopenia, recurrent infections |
| Metabolic | poor feeding, vomiting, disturbance of consciousness, enlarged liver, metabolic acidosis, hyperammonemia, hyperkalemia, hyperhomocysteinemia, hypotonia, metabolic acidosis, abnormal electroencephalogram, anemia, cardiomyopathy, metabolic alkalosis, hyperphenylalaninemia, physical retardation, lethargy, seizures, coma, poor growth, intrahepatic cholestasis, increased serum citrulline |
| Musculoskeletal | multiple osteochondroma, open skull sutures, hypoplasia of the clavicles, shortened limbs, congenital laryngomalacia, small chest, scoliosis, clubfoot of the equinovarus type (talipes equinovarus), abducted thumbs and great toes, and cleft palate |
| Dermatologic | dry skin，desquamation of skin，wrinkled skin，ocular hypertelorism, high palatal arch |
| Audiologic | hearing loss |
| multiple organs | polycystic kidney, cystic nodules of the liver, respiratory failure, atrial septal defect, poor feeding, strabismus, hypomyotonia, thin skin, craniofacial anomalies, skeletal anomalies, facial dysmorphism，prenatal and postnatal growth retardation, upper limb anomalies, global developmental delay |
